# Partial mitochondrial genome of the Sanchiang Tree Toad *Hyla sanchiangensis* (Anura: Hylidae)

**DOI:** 10.1080/23802359.2020.1787890

**Published:** 2020-07-07

**Authors:** Qiao-Er Chen, You-Fu Lin, Li Ma, Guo-Hua Ding, Zhi-Hua Lin

**Affiliations:** a Laboratory of Amphibian Diversity Investigation, College of Ecology, Lishui University, Lishui, China; b College of Forestry, Nanjing Forestry University, Nanjing, China

**Keywords:** *Hyla sanchiangensis*, mitochondrial genome, phyolgentic relationship, next-generation sequencing

## Abstract

We reported the partial mitochondrial genome (mitogenome) for *Hyla sanchiangensis* (Anura: Hylidae), a arboreal frog and endemic in China. The length of partial mitogenome of *H. sanchiangensis* was 15,664 bp, and contained PCGs (COX1-3, ND1-6, ND4L, ATP6, ATP8 and CYTB), 2 ribosomal RNA genes, 22 transfer RNA genes, and 2 non-coding regions of a L-strand replication origin and a partial loop region. The overall base composition of the sequence is 29.91% A, 29.86% T, 14.58% G, and 25.65% C, with a total A + T content of 59.77%. The result of phylogenetic analysis showed that *H. sanchiangensis* formed a clade with other species belonging to the genus of *Hyla*. This mitogenome data could help in evolutionary biology and population genetics of the Hylid species.

There are currently 17 species of *Hyla* (Anura: Hylidae) distributed in Eurasia (Frost 2020). Eight species of *Hyla* are currently known in China, and six of which are endemic to China (AmphibiaChina 2020). The Sanchiang Tree Toad (*Hyla sanchiangensis*) is a endemic Chinese arboreal frog, which is distributed in southeastern of China (AmphibiaChina 2020). At GenBank, only three mitochondrial genomes (mitogenomes) are available for *Hyla* speices. Here, we attempted to sequence the complete mitogenome of *H. sanchiangensis* via next-generation sequencing, and retrieved successfully the genetic information about partial mitogenome of the above mentioned species.

The specimen (LSU20200425002JL) of *H. sanchiangensis* was collected in Jiulongshan National Nature Reserve (28.37°N, 118.90°E), Zhejiang, China, and deposited in the Museum of Laboratory of Amphibian Diversity Investigation (ADI) at Lishui University. Total DNA was extracted from muscle tissue of *H. sanchiangensis* using EasyPure Genomic DNA Kit (TransGen Biotech Co, Beijing, China). The mitogenome of *H. sanchiangensis* was acquired by Illumina NovaSeq 6000 (Novogene Bioinformatics Technology Co. Ltd., Tianjin, China) for PE 2 × 150 BP sequencing. Raw sequence data (16.25 G) was deposited in NCBI’s Sequence Read Archive (SRA; accession: SRR11921085). We used the NOVO Plasty 3.7 to *de novo* assembled the clean data without sequencing adapters (Dierckxsens et al. 2017).

This study reported a 15,664 bp long sequence of *H. sanchiangensis* partial mitogenome (GenBank accession: MT561180), which containing 13 PCGs (COX1-3, ND1-6, ND4L, ATP6, ATP8 and CYTB), 2 ribosomal RNA (rRNA) genes, 22 transfer RNA (tRNA) genes, and 2 non-coding regions of a L-strand replication origin and a partial loop region. The overall base composition of the sequence is 29.91% A, 29.86% T, 14.58% G, and 25.65% C, with a total A + T content of 59.77%. Similar to the mitogenomes of other *Hyla* species, the AT content is higher than the GC content (Zhang et al. 2005; Kang et al. 2016; Ye et al. 2016). The H-strand encodes12 PCGs, 14 tRNA genes, and 2 rRNA genes, while the ND6, and 8 other tRNA genes are encoded on the L-strand. Among the 13 PCGs, the ND5 was the longest (1803 bp), while the ATP8 was the shortest (165 bp). Codon usage analysis of H. sanchiangensis showed that three kinds of start codons (ATG, ATT, and TTG) and four kinds of stop codons (TAA, AGA, TAG, and T) were used. The 12S rRNA (937 bp) and 16S rRNA genes (1596 bp) are located between tRNA^Phe^ and tRNA^Val^ and between tRNA^Val^ and tRNA^Leu^, respectively. The 22 tRNA genes varied in size from 64 to 73 bp.

Phylogenetic analysis was inferred from available mitogenomes of *H. sanchiangensis* and other 9 Hylid species based on 13 PCGs with *Bufo gargarizans* (Anura: Bufonidae) as the outgroup using Bayesian inference (BI) methods. The optimal substitution model (GTR + I + G) was implemented via jModelTest (Darriba et al. 2012). Four parallel runs of Markov Chain Monte Carlo (MCMC) were analyzed for 1,000,000 generations, sampling every 1000 generations and discarded 1000 trees as burn-in. The results of phylogenetic relationship showed that *H. sanchiangensis* formed a clade with other species (*H. annectans*, *H. chinesis*, and *H. tsinlingensis*) belonging to the genus of *Hyla*, the genus *Hyla* was closely related with the genus *Dryophytes* than to other genera, and the genus *Pseudis* was a basal clade relative to others within Hylidae (Figure 1). This partial mitogenome sequence of *H. sanchiangensis* could help in evolutionary biology, population genetics of the Hylid species.

**Figure 1.**
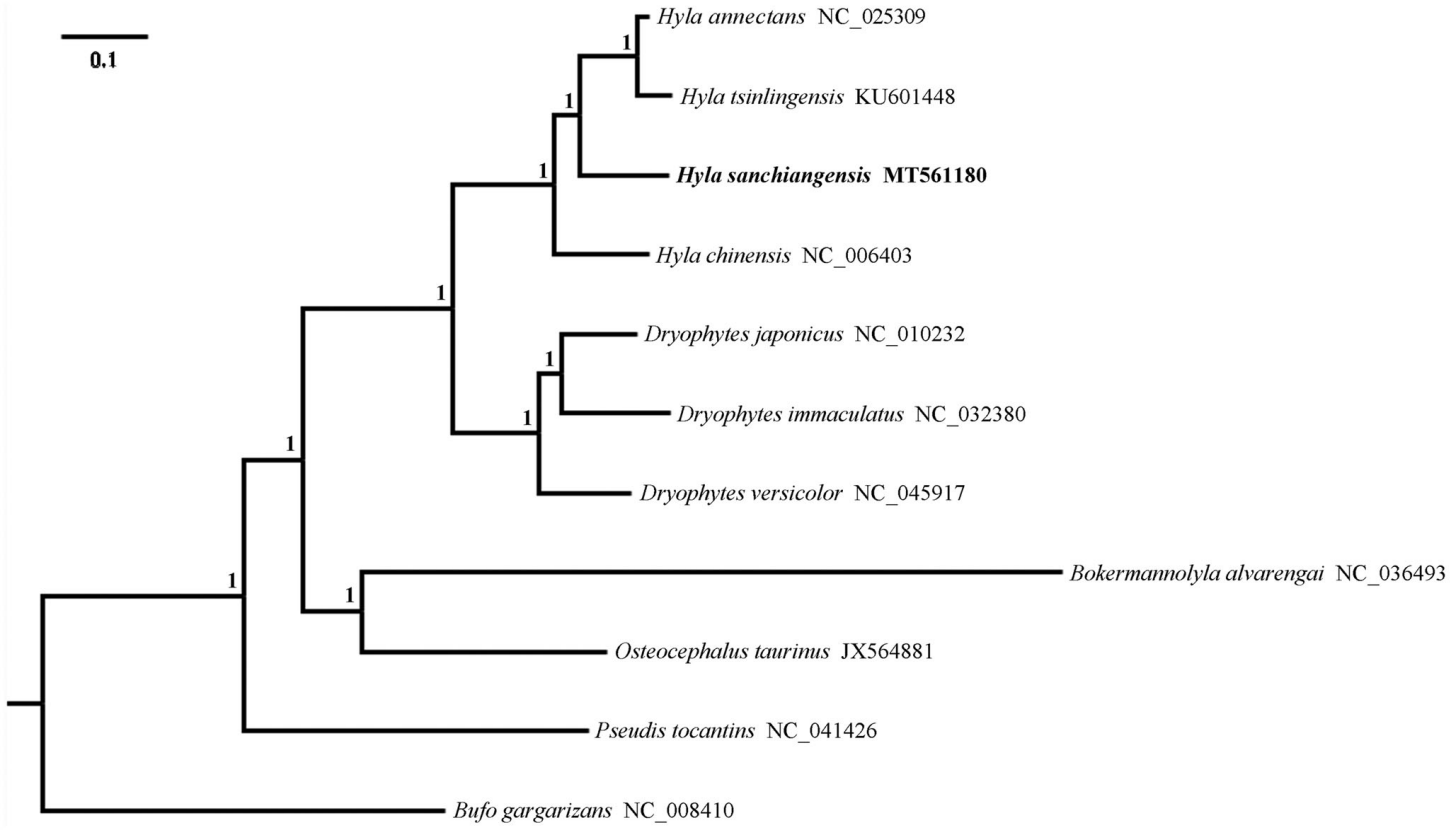
The phylogenetic tree obtained from the BI analysis, based on 13 concatenated mitochondrial PCGs. Numbers on the node are posterior probability.

## Data Availability

The data that support the findings of this study are openly available in NCBI at www.ncbi.nlm.nih.gov reference number [MT561180].
